# The Effect of Music on the Human Stress Response

**DOI:** 10.1371/journal.pone.0070156

**Published:** 2013-08-05

**Authors:** Myriam V. Thoma, Roberto La Marca, Rebecca Brönnimann, Linda Finkel, Ulrike Ehlert, Urs M. Nater

**Affiliations:** 1 Department of Psychology, Brandeis University, Waltham, Massachusetts, United States of America; 2 Clinical Psychology & Psychotherapy, University of Zürich, Zürich, Switzerland; 3 Clinical Biopsychology, University of Marburg, Marburg, Germany; Pennington Biomedical Research Center, United States of America

## Abstract

**Background:**

Music listening has been suggested to beneficially impact health via stress-reducing effects. However, the existing literature presents itself with a limited number of investigations and with discrepancies in reported findings that may result from methodological shortcomings (e.g. small sample size, no valid stressor). It was the aim of the current study to address this gap in knowledge and overcome previous shortcomings by thoroughly examining music effects across endocrine, autonomic, cognitive, and emotional domains of the human stress response.

**Methods:**

Sixty healthy female volunteers (mean age = 25 years) were exposed to a standardized psychosocial stress test after having been randomly assigned to one of three different conditions prior to the stress test: 1) relaxing music (‘*Miserere*’, Allegri) (RM), 2) sound of rippling water (SW), and 3) rest without acoustic stimulation (R). Salivary cortisol and salivary alpha-amylase (sAA), heart rate (HR), respiratory sinus arrhythmia (RSA), subjective stress perception and anxiety were repeatedly assessed in all subjects. We hypothesized that listening to RM prior to the stress test, compared to SW or R would result in a decreased stress response across all measured parameters.

**Results:**

The three conditions significantly differed regarding cortisol response (*p* = 0.025) to the stressor, with highest concentrations in the RM and lowest in the SW condition. After the stressor, sAA (*p*=0.026) baseline values were reached considerably faster in the RM group than in the R group. HR and psychological measures did not significantly differ between groups.

**Conclusion:**

Our findings indicate that music listening impacted the psychobiological stress system. Listening to music prior to a standardized stressor predominantly affected the autonomic nervous system (in terms of a faster recovery), and to a lesser degree the endocrine and psychological stress response. These findings may help better understanding the beneficial effects of music on the human body.

## Introduction

Prolonged experiences of stress are related to poor individual health [1,2] and associated with substantial financial costs for the society [3]. As a result, the development of cost effective stress prevention or stress management approaches has become an important endeavor of current research efforts. Music has been shown to beneficially affect stress-related physiological [4–6], as well as cognitive [7], and emotional processes [8,9]. Thus, the use of listening to music as an economic, non-invasive, and highly accepted intervention tool has received special interest in the management of stress and stress-related health issues.

The experience of stress arises when an individual perceives the demands from the environment ‘…as taxing or exceeding his or her resources and endangering his or her well-being' [10]. Accordingly, physiologic stress effects are regulated by top-down central nervous system processes (=*cognitive* stress component, e.g. ‘I can’t cope with the situation’), as well as by sub-cortical processes within the limbic system (=*emotional* stress component, e.g. ‘anxiety’). Both areas forward their messages (e.g. ‘I am in danger!’) via neuronal pathways to a central control system, the hypothalamus [11]. The hypothalamus is closely intertwined with two major stress systems, the hypothalamus-pituitary-adrenal (HPA) axis and the sympathetic nervous system (SNS) (=*physiologic* stress component, i.e. endocrine and autonomous responses). Together, the HPA axis and the SNS orchestrate various psychological (e.g. emotional processing) and physiological (e.g. endocrine and cardiovascular activation) processes to ensure the maintenance of the homeostasis of the organism that is challenged by the experience of stress [11–13]. The main effector of the HPA axis is the so-called ‘stress’ hormone cortisol; its concentration is measured and evaluated in order to have an index for HPA axis activation [14,15]. Salivary alpha-amylase (sAA) is a novel biochemical index for sympathetic nervous system (SNS) activity [16–19]. Both parameters obtained particular interest in stress research as unlike more traditional blood-derived stress markers (e.g. epinephrine and norepinephrine), they can conveniently be assessed in saliva. Taken together, the experience of stress is a multi-faceted phenomenon that comprises cognitive and emotional components that are closely intertwined with physiological systems, whose messengers / effectors found in saliva can be applied to objectively measure stress responses.

Research on potentially beneficial effects of music listening on HPA axis functioning, i.e. on stress-induced cortisol release, has only recently been established. Significant positive changes in cortisol were reported when listening to music before and / or during medical interventions considered stressful (decreases and lower increases in cortisol) [20–22] and after such interventions (greater reductions in cortisol) [23,24]. The few laboratory-based studies show inconsistent findings, though: some report that music was effective in suppressing a stress-related increase in cortisol [25], or in decreasing cortisol levels following a stressor when compared to a non-music control condition [5]. However, some other investigations did not find a meaningful impact of music on cortisol [26,27]. As a consequence, no final conclusions can be drawn about whether or how music listening influences stress-induced cortisol levels.

The research on beneficial effects on SNS parameters has a longer tradition: A series of clinical and laboratory-based studies revealed that listening to music can decrease sympathetic activity [28–30]. However, positive SNS effects of listening to music are not consistently reported [30,31]. It is conceivable that knowledge achieved from the effects of music on an additional SNS parameter, such as the newly established sAA, would help to increase understanding of inconsistent previous reports. However, to date no laboratory study exists that has investigated the effects of music on stress-induced sAA levels.

As listening to music has the capacity to initiate a multitude of cognitive processes in the brain [32], it might be assumed that music also influences stress-related cognitive processes and, as a consequence, physiological responses. Previous investigations found reductions in perceived levels of psychological stress, increased coping abilities, or altered levels in perceived relaxation after listening to music in the context of a stressful situation [7,33]. Another line of research has focused on the effects of music on anxiety, which may be considered an adaptive response to the experience of stress. Given that music listening can trigger activity in brain regions linked to the experience of (intense) emotions [8,34–36], listening to music might also modulate anxiety levels induced by the experience of stress. Indeed, a decrease in anxiety after listening to music is the most consistent findings reported in field studies with patients [22,37,38] and laboratory-based studies [26,39]. Nevertheless, not all investigations found anxiety reductions through music listening [40–42]. Also here, no final conclusions can be drawn whether or how music is able to influence cognitive and emotional components of the stress response.

Besides the insufficient quantity or pure lack of studies investigating the effects of music on stress-induced cortisol or sAA levels, there are a number of methodological limitations that may account for the wide discrepancies in the already existing literature. The main reason for this divergence in the literature might be that many studies have been conducted in a clinical context, introducing heterogeneity by studying various different medical settings and patient samples. Besides the valuable attempt of a small number of studies to investigate the effect of music listening in a controlled laboratory environment, these investigations suffered from methodological shortcomings, such as small sample size [5] and/or the lack of a valid (i.e. HPA axis activating) stressor. From the perspective of biopsychological stress research, a major shortcoming is the vast neglect of the control of confounding variables [25–27]. Although acute stress responses occur rather uniformly across individuals (which makes it a good paradigm for the investigation of acute stress), they may be modified by previous individual experiences, such as chronic (affective) stress [43,44]. What is more, given that stress [45], as well as music behavior [46,47] differs as a matter of how emotions are regulated in general, traits of emotion regulation should always be controlled, particularly in investigations examining the effect of music on stress. Finally, the broad majority of previous work has used only one (if any) control group (rest with no acoustic stimulation), and has not examined whether positive effects of music are due the nature of music itself or due to a calming (non-music) acoustic stimulation. Consequently, the particular effectiveness of music listening on stress cannot yet be determined.

In sum, it appears that listening to music has the inherent ability to decrease the psychobiological stress response. However, due to the fact that the existing literature is not complete and often appears as inconsistent, definitive conclusions about the beneficial stress-reducing effect of music may be too premature. In light of these considerations, we set out to examine the effect of listening to music prior to a standardized stressor across neuroendocrine, autonomic, cognitive, and emotional domains of the human stress response in healthy participants in a laboratory setting. We put a special emphasis on the control of known influencing factors of the stress response and music effects, i.e. depression, anxiety, chronic stress, and emotion regulation traits. To the best of our knowledge, such an endeavor has not been attempted thus far. We hypothesized that those participants who listened to relaxing music prior to the stress task would show a different stress responses in terms of cortisol, salivary alpha-amylase, heart rate, respiratory sinus arrhythmia, subjective perception of stress, and anxiety when compared to non-music control groups, i.e. an acoustic control condition (sound of rippling water) and a control condition resting without acoustic stimulation.

## Methods

### Participants

**Figure 1:**
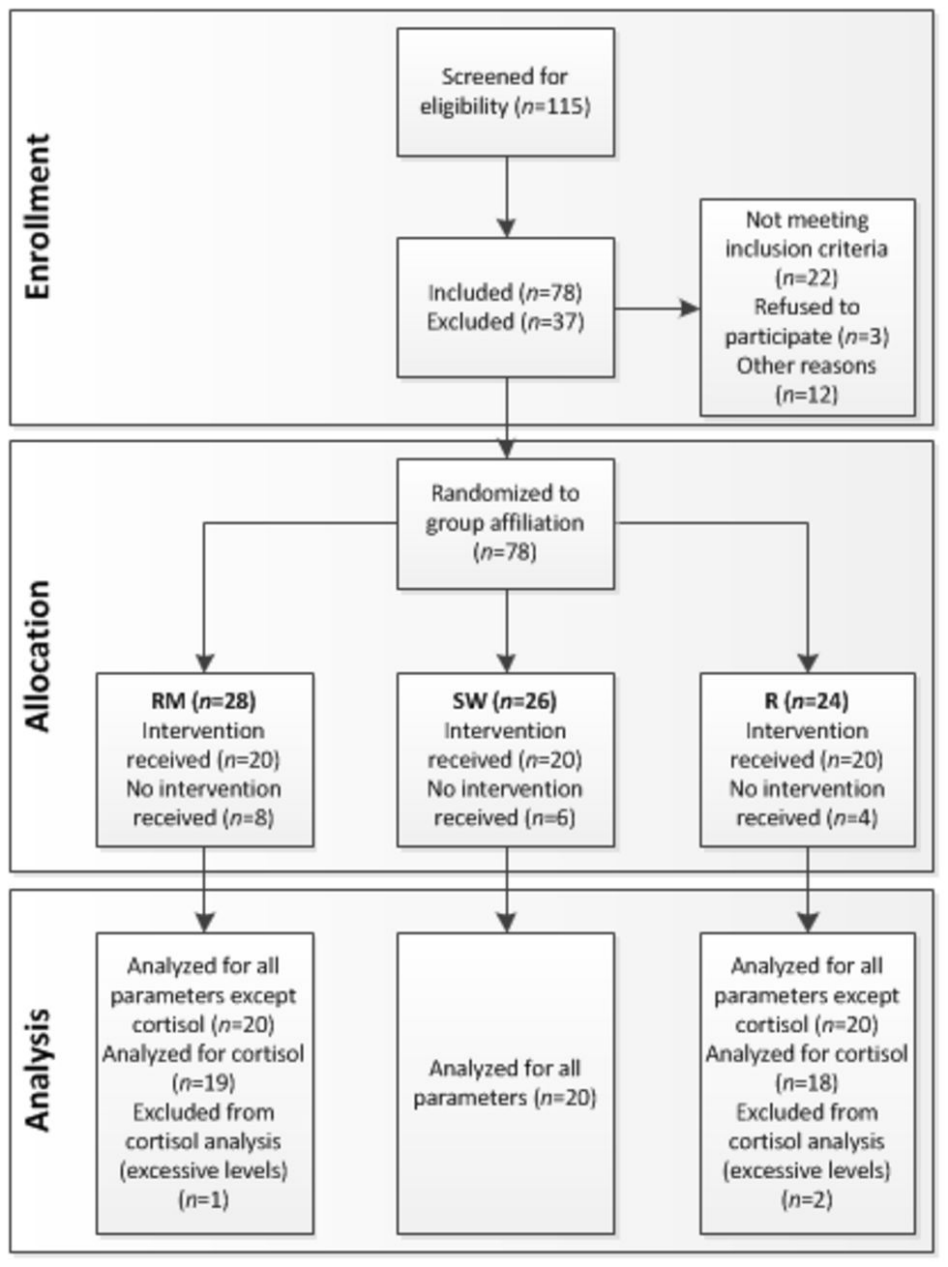
Flow diagram. Flow diagram of the process through the phases of enrollment, allocation and analysis.

Participants were recruited by advertisement at the University of Zurich and the Swiss Federal Institute of Technology, Zurich (Figure 1). In a telephone screening, criteria for eligibility of interested participants (female sex, BMI between 18–25 kg/m^2^, 20–30 years of age, [Swiss] German as native language and a regular menstrual cycle) were verified. Female sex was chosen to control for gender differences, as sexual dimorphism in both the HPA axis response to psychosocial stress [48,49] and in physiological and emotional responses to 9 music listening [6,29,50] have been observed in the past. Given their confounding effect on the organism in general, and the HPA axis in particular, exclusion criteria of the current study were the following; current depression, self-reported acute and chronic somatic or psychiatric disorders, medication, use of hormonal contraceptives, use of psychoactive substances, and excessive consumption of alcohol (> 2 alcohol beverages / day) or tobacco (> 5 cigarettes / day). Additionally, self-reported hearing deficits or tinnitus were exclusion criteria. Individuals with musical training were not included in the study. If eligibility requirements were met, and oral agreement was obtained, appointments were scheduled during the woman’s follicular phase (days 4-10) of the menstrual cycle to control for hormonal variation throughout the menstrual cycle.

In advance of the appointment, participants were sent a set of information and several questionnaires (see below). In the advance material, participants were informed about the course of the study, but were not given detailed information about the experimental stress paradigm. Study language was (Swiss) German. Participants were instructed not to drink alcohol or caffeinated beverages 48 hours prior to the study. Additionally, they were told to refrain from any exercise activities 24 hours prior to the experiment. Further, participants were asked to refrain from brushing their teeth or eating at least 60 minutes before the study. For their participation in the study, the participants were reimbursed with 50 Swiss Francs.

An a priori power analysis was conducted to estimate the optimal sample size to answer the main hypothesis of a decreased cortisol response in the music group when compared to the control groups. It indicated that 54 participants were required to reach an 87% power for detecting an effect of 0.15 when employing an alpha criterion of 0.05 of statistical significance.

### Ethics Statement

The study was conducted in accordance with the Declaration of Helsinki. The study protocol was approved by the ethics committees of the University of Zurich and of the Canton of Zürich. Oral and written informed consent from all subjects was obtained.

### General procedures

#### Study design

The experiment used a *between subject* design to compare the effect of acoustic stimulation (independent variable) on cortisol, sAA, HR, RSA, mood, and anxiety (dependent variables). There were three conditions prior to a stress test (Trier Social Stress Test, TSST, see description below): a music condition (relaxing music listening prior to stress test, RM), a water sound condition (an acoustic control condition including listening to sound of rippling water, SW) and a control condition (non-acoustic control condition including resting without acoustic stimulation, R). Seventy-eight participants fulfilled all study requirements and were randomly assigned to one of the groups. Eighteen participants were not able to keep their appointment (see Figure 1). Randomization was accomplished through the use of a computer generated randomization list.

#### Psychobiological stress induction

All participants underwent a standardized psychosocial laboratory stress protocol. The TSST consists of an introduction (Intro) that lasts 2 minutes in which participants are introduced to the procedure of the TSST. Specifically, they are told that the TSST consists of a public speaking task followed by a mental arithmetic task in front of an audience. In the public speaking task (lasting 5 minutes), participants are asked to apply for a job. In this simulated job interview, they are asked to talk about their personal qualifications for the chosen job, e.g. why they are a better fit for the job than other applicants. Right after the job interview, participants are explained the nature of the mental arithmetic task, which lasts for another 5 minutes. The participants have to calculate backwards in steps of 17 from the number 2043. After each calculation error the participants are asked to re-start calculating from 2043. The TSST has repeatedly been found to be a reliable tool to activate both the HPA axis and the autonomous nervous system (ANS) [51]. In the current study, the standard TSST procedure as reported in the literature was slightly modified: in the Intro, the subjects were not told about the exact nature of the upcoming speaking task (i.e. giving a speech as part of a simulated job interview) in order to prevent subjects from mentally preparing for the task.

#### Study procedure

**Figure 2:**
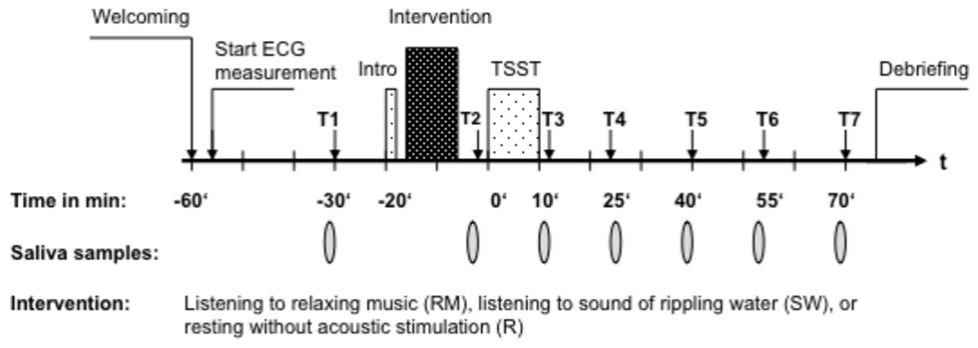
Study Procedure. Timeline of the testing procedure.

For the current study, all examinations were conducted between 1200 and 1700h to minimize the confounding effect of the hormonal diurnal rhythm. Circadian fluctuations of hormone levels are particularly pronounced in the morning hours and flatten throughout the day [52,53]. Participants arrived at the laboratory 60 min prior to the onset of the stress induction by the TSST (Figure 2). Participants were then escorted to a non-intervention room, where they spent their waiting time between the actual experimental interventions. Immediately after arrival, participants were informed by the main experimenter about the course of the experiment. Oral and written informed consent was obtained from all participants. Right afterwards, the LifeShirt, an electrophysiological measurement device (see below), was attached. After an adaptation period of 30 min, a basal saliva sample (T1, -30 min) was taken. Twenty minutes prior to the TSST, the participants were brought to the TSST room, where they were introduced by the main experimenter to the procedure of the TSST (= introduction: Intro, 2 min). The subjects were then brought to the intervention room, seated in a comfortable chair, and provided with headphones. All participants had to adjust a test signal (sinus tone, sound pressure = -70dB) to the individual hearing threshold level for the calibration of the volume. After this, the participants were to undergo their assigned condition, i.e. RM, SW, or R for ten minutes. No instruction was given for any of the conditions. Immediately after this part a second saliva sample was taken (T2, -5 min). Following this, subjects were taken back into the TSST room where they were undergoing the TSST. After the completion of the TSST, the subjects were then returned back the non-intervention room and a third saliva sample was taken (T3, + 10 min). Further samples were taken 15 min (T4, + 25 min), 30 min (T5, + 40 min), 45 min (T6, + 55 min), and 60 min (T7, + 70 min) after the TSST. In addition, the subjects completed various self-report stress measures (see below) at T1, before and after T2, at T3 and T4.

#### Music stimulus and acoustic control stimulus

‘*Miserere*’ by Allegri (CD Gimell 454 939-2) is a soothing and calming music piece (Latin choral singing) that was chosen to induce relaxation in our subjects. The stimulus was selected on the basis of previous research [6]. We decided to use a single standardized music stimulus, as this approach is thought to have a greater effect on stress reduction than music stimuli selected by the subjects themselves [54]. Further, we wanted to avoid possible influences of memory or subjective associations with self-chosen music stimuli by participants.

We included a non-music acoustic control condition, i.e. listening to sound of rippling water, in our study. This control condition has been chosen to control for effects on psychological and physiological parameters, which might be caused by mere acoustic stimulation alone. The sound of rippling water is missing the typical characteristics of music, such as a structured melody and rhythm. Still, it is an acoustic stimulus with a certain perceptual quality for the listener. What is more, in comparison to artificially produced sounds (such as white or pink noise or single tones), the sound of rippling water may be presented for longer periods of time without exerting stress or boredom in the listener [50].

### Measures

#### Electrophysiological and biochemical measurement and analyses

Heart rate (HR) and respiratory sinus arrhythmia (RSA) were measured with the LifeShirt® System, an ambulatory detection system that allows the continuous monitoring of cardiorespiratory parameters [55], and edited manually to correct for ectopic beats with the VivoLogic 3.1 software (Vivometrics, Ventura, CA, USA). RSA is a measure for variations in HR within a breathing sequence; it is used as an indicator for parasympathetic cardiac control. HR and RSA were determined for 5-minute segments, ranging from a baseline interval prior to the Intro until 30 minutes after completion of the TSST.

For the analysis of cortisol (as an indicator of HPA axis activity) [15] and salivary alpha-amylase (sAA, as an indicator of autonomic activity) [16,17], saliva was collected using small cotton swabs (Salivettes, Sarstedt, Sevelen, Switzerland). Stimulated saliva was taken by having the participants gently chewing the cotton roll for 1 min. Thereafter, the cotton roll was placed into a small plastic tube. Samples were stored at -20° C until biochemical analysis took place. Salivary free cortisol was determined by using a commercial chemiluminescence immunoassay (LIA) (IBL, Hamburg, Germany). Inter- and intraassay coefficients of variation were below 10%. All samples of one subject were analyzed in the same run to reduce error variance caused by imprecision of the intraassay. Activity in sAA was analyzed using the microplate reader Synergy HT Multi-Mode (BioTek) and adapted assay kits obtained from Roche. The assay is a kinetic colorimetric test. Inter- and intraassay variance was below 1%.

#### Psychometric measurements

Demographic information such as age, education, medication intake, nicotine use and illnesses were collected using a demographic questionnaire. Questionnaires were used to investigate the role of music preference and psychological factors.

The Music Preference Questionnaire (MPQ) [56] was used to assess participants’ general preference for Classical music, also in relation with their general music preference for the most common music styles: Pop, Rap / Hip Hop, Latin, Soul / Funk, Hard Rock, Electro, New Age, Country, and Jazz music. On a 5-point Likert scale participants indicated how much they liked the particular music style (1 ‘not at all’ to 5 ‘very much’).

The Beck Depression Inventory (BDI) [57] was used to control for a possible impact of depression on the HPA axis response [58]. Scores higher than 18 are suggestive of clinically relevant depression.

Depending on the dispositional preferred emotion regulation strategy, different cognitions, emotions, and behavior may result in and after emotional situations. To control for the impact of how emotions are regulated in general the validated German version [59] of the Emotion Regulation Questionnaire (ERQ) by Gross and John [60] was used. The ERQ assesses two common trait emotion regulation strategies, *reappraisal* and *suppression*. Higher values on each scale denote greater expressiveness of the respective variable.

Visual analog scales (VAS) were employed to repeatedly measure subjective perception of stress during the experiment. To control for the experience of chronic stress in our sample, we used the screening scale of the *Trier Inventory for the Assessment of Chronic Stress* (TICS) [61], which assesses the global perceived chronic stress load of an individual with 12 items (Screening Scale of Chronic Stress, SSCS). Participants were required to rate how often they had experienced certain stressful situations during the past three months on a 5-point Likert scale. High values are indicative that the individual is often worried, overburdened, overstrained, and unacknowledged.

The State and Trait Anxiety Inventory (STAI) [62] was used to assess anxiety. The STAI consists of two 20-items questionnaires which assess state respectively trait levels of anxiety in clinical and non-clinical populations. Scores for both scales range between 20 (low anxiety) and 80 (high anxiety). The STAI-state was used as a continuous measurement for possible changes in anxiety during the experiment. The STAI-trait was used to control for the effect of anxiety as a personality trait in our sample [63].

The stimuli questions were used to assess the subjective perception of either music or sound of rippling water. Subjects were required to rate how much they liked the stimulus, and how relaxing they perceived the stimulus on a 5-point Likert scale immediately after the stimulus presentation. High values are indicative for increased liking and of an increased relaxing effect of the stimulus.

### Statistical analysis

Data analyses were performed using SPSS (17.0) software packages (SPSS, Chicago, IL, USA). Homogeneity of variance was tested using Levene’s test before statistical analyses were applied. All reported results were corrected by the Greenhouse-Geisser procedure where appropriate (violation of sphericity assumption) [64,65]. In case of missing data, cases were excluded list wise. Analyses of variance (ANOVAs) for repeated measures were computed to analyze possible time, condition and interaction effects. For comparison of the scale means of the questionnaires with normative samples, Student’s t-tests were computed. Cortisol (-30 min to + 70 min), alpha-amylase levels as well as heart rate measures (-30 min to + 40 min) were evaluated according to the area under the curve with respect to increase (AUC_I_). The AUC_I_ is related to the sensitivity of the biological system; it is pronouncing changes over time, and is characterized by accumulation of the error of the baseline, as the formula is based on the difference between the baseline and the subsequent measures [66]. To estimate the extent of stress reactivity of cortisol, sAA, HR, and RSA, we calculated the delta measures of the stress responses (peak values after stressor minus baseline values before stressor), and refer to it as *peak delta*. For the estimation of a recovery value, we subtracted the first baseline value after the stressor from the peak values after the stressor (delta), and refer to it as *recovery delta*. Calculated measures of AUC_I_, peak delta and recovery delta were analyzed using ANCOVAs. For all analyses, results were considered statistically significant at the *p* ≤ 0.05 level, and were considered a trend at the *p* < 0.1 level. All tests were two-tailed. Unless indicated otherwise, all results shown are means ± standard deviations (SD).

## Results

### Sample characteristics

Sixty healthy female subjects participated in the study (age mean = 25.3 years, SD = 3.21 years; BMI (calculated as weight in kilograms divided by the square of height in meters) mean = 21.63, SD = 2.34; years of education mean = 15.3, SD = 2.56). Mean BDI scores of 5.5 (SD = 3.0; range = 0 - 17) indicate no clinically significant depressive symptom severity in our sample. Mean TICS summary scores of 17.32 (SD = 7.55; range = 0 - 36) indicate low levels of chronic stress load in our sample. Mean STAI-trait scores of 37.47 (SD = 9.86; range = 22–63) indicate low trait anxiety levels in our sample [see 62]. Regarding trait emotion regulation strategies, the mean scores of the two scales of the ERQ (*reappraisal* mean 4.83, SD = 1.0; *suppression* mean 3.29, SD = 1.08) were in a comparable range to the female norm sample described by Gross and John [60]. Music preference for classical music was high in the current sample; only Pop music was preferred more (Classical music preference mean = 3.33, SD = 1.1; Pop music preference mean = 3.72, SD = 1.15). Randomization resulted in 20 participants undergoing the experimental condition (RM), 20 participants undergoing the non-music acoustic control condition (SW), and 20 participants undergoing the control condition without acoustic stimulation (R). The randomized assignment to groups was evaluated by comparing demographic variables (age, BMI, years of education), preference for Classical music, and means of all control variables (BDI, ERQ, STAI-trait, TICS) between groups (all *p* = n.s.) (TABLE 1). We found no significant differences between groups.

**Table 1:** Sample characteristics (Means, standard deviations, and group differences among study variables).

**Characteristic**	**RM (*n*=20)**	**SW (*n*=20)**	**R (*n*=20)**	***p*-valueª**
Age (years)	25.13 (2.86)	25.0 (3.71)	25.67 (3.13)	0.79
Years of education	15.32 (2.26)	14.94 (2.41)	15.61 (3.05)	0.75
MP Classical music	3.05 (1.32)	3.5 (1.05)	3.45 (0.89)	0.37
BMI	21.28 (2.08)	21.83 (2)	21.79 (2.9)	0.71
BDI	4.84 (3.56)	5.63 (4.21)	6.0 (4.01)	0.65
ERQ reappraisal	4.75 (0.89)	4.98 (0.98)	4.76 (1.15)	0.71
ERQ suppression	3.0 (1.07)	3.68 (0.93)	3.19 (1.17)	0.13
STAI-trait	35.8 (8.91)	38.35 (10.87)	38.25 (9.98)	0.66
TICS	16.75 (7.77)	16.05 (8.92)	19.1 (5.75)	0.42

n = valid cases, RM = listening to relaxing music, SW = listening to sound of rippling water, R = resting without acoustic stimulation, MP = Music preference, BMI = Body mass index, BDI = Beck Depression Inventory, ERQ = Emotion Regulation Questionnaire, STAI = State and Trait Anxiety Inventory, TICS = Trier Inventory for the Assessment of Chronic Stress

ª Probability value from one-way ANOVA.

### Stimuli characteristics

Participants liked both acoustical stimuli (music: mean=3.21; SD=1.36; sound of rippling water: mean=3.84; SD=1.17) and both were perceived as relaxing (music: mean=4.0; SD=0.88; sound of rippling water: mean=4.0; SD=1.2). None of our participants in the acoustic stimulation groups expressed any negative comments about the stimuli (music / sound of rippling water).

### Salivary cortisol responses

**Figure 3:**
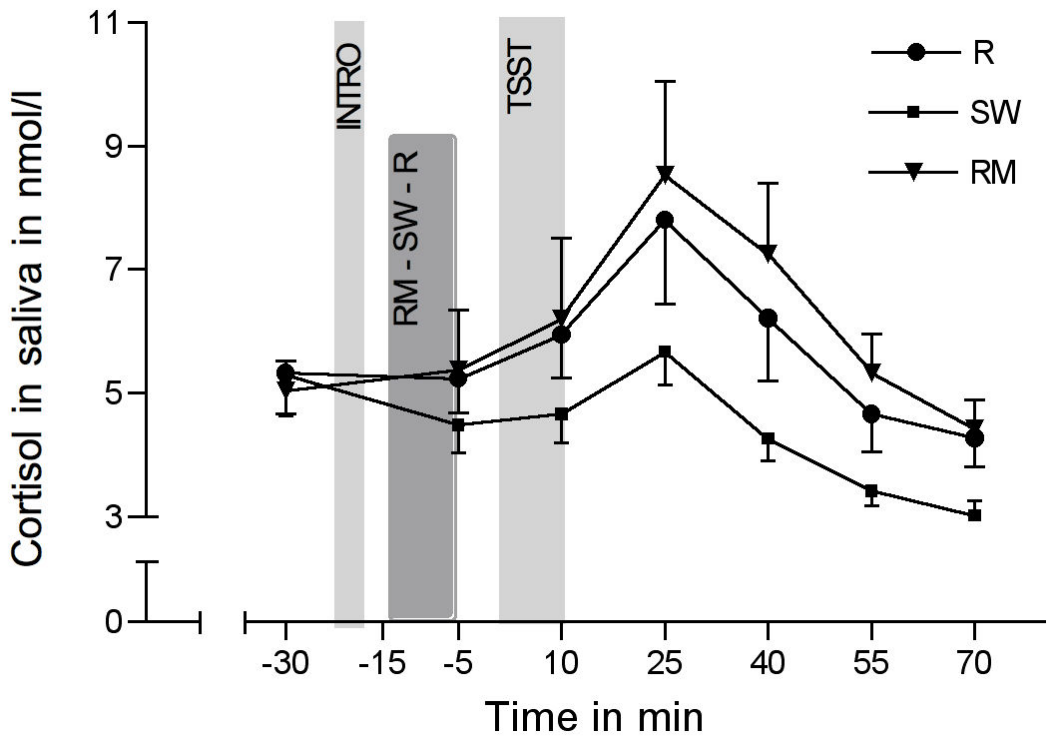
Salivary cortisol levels in response to the TSST. Salivary cortisol levels in response to the TSST (means ± SEM), in the experimental group listening to relaxing music (RM), the control group listening to sound of rippling water (SW), and the control group resting without acoustic stimulation (R).

One participant of the RM condition and two participants of the R condition showed levels of cortisol that were more than 3 standard deviations higher than the mean. As a consequence, these participants were excluded from all further analyses. The stress protocol induced significant increases in salivary cortisol in all groups over time (*F*(2.48/133.74)=18.46; *p*<0.001; *η* ^2^=0.255). Without including the control variables, repeated-measures ANOVA revealed no significant group differences (group-by-time interaction: *F*(4.95/133.74)=1.67; *p*=0.146; *η* ^2^=0.058). With the inclusion of the control variables (BDI, ERQ, STAI-trait, TICS), cortisol concentrations differed significantly between groups, with highest values in the RM and lowest values in the SW (group-by-time interaction: *F*(5.05/116.13)=2.68; *p*=0.025; *η* ^2^=0.104) (Figure 3). Single controlled group comparisons revealed a significant difference between the RM and SW groups (group-by-time interaction: *F*(2.33/67.6)=4.72; *p*=0.009; *η* ^2^=0.140), but not between RM or SW when compared to R (all n.s.). Finally, univariate analyses revealed a significant difference between RM and SW in their peak delta (*F*(1/29)=5.18; *p*=0.03; *η* ^2^=0.152), and in the AUC_I_, again between the groups RM and SW (*F*(1/29)=4.7; *p*=0.039; *η* ^2^=0.139). No significant differences were found when RM and SW were compared to R regarding peak delta and AUC_I_.

### Salivary alpha-amylase responses

**Figure 4:**
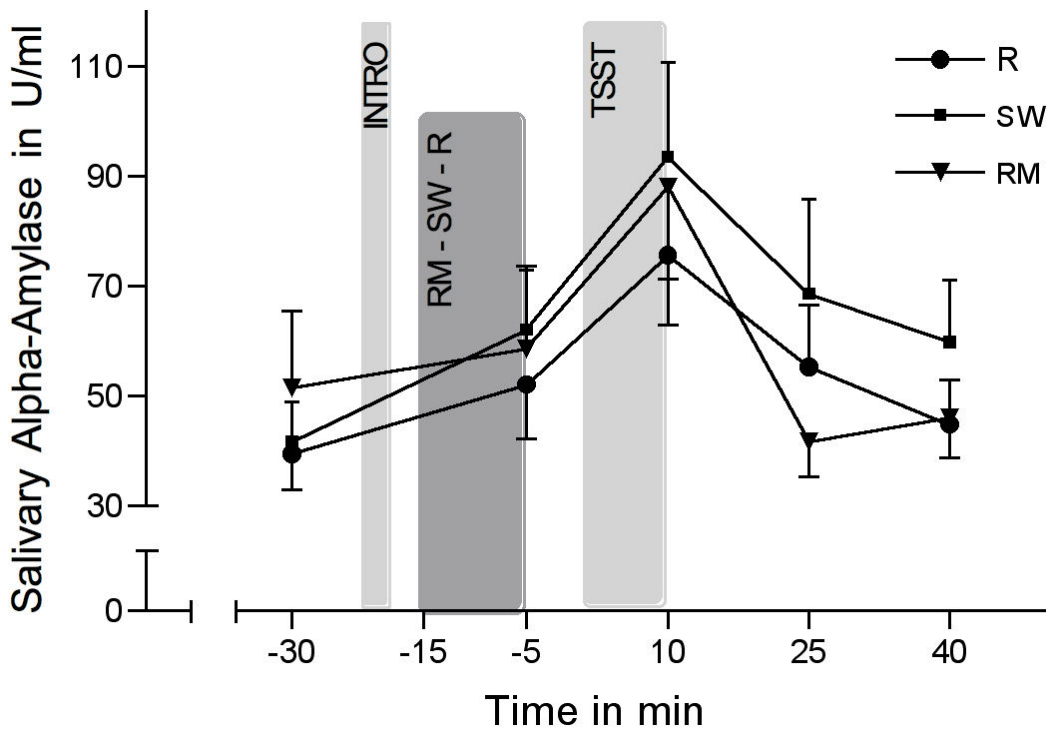
Salivary alpha-amylase activity in response to the TSST. Salivary alpha-amylase activity in response to the TSST (means ± SEM) in the experimental group listening to relaxing music (RM), the control group listening to sound of rippling water (SW), and the control group resting without acoustic stimulation (R).

sAA activity increased significantly over the course of the stress task (*F*(2.62/146.82)=15.60; *p*<0.001; *η* ^2^=0.218). Without the inclusion of the control variables (i.e. BDI, ERQ, STAI-trait, TICS), repeated-measures ANOVA revealed no significant group differences (group-by-time interaction: *F*(5.24/146.82)=1.19; *p*=0.318; *η* ^2^=0.041). Also with the inclusion of the control variables, we found were no significant differences in sAA activity between groups (group-by-time interaction: *F*(4.96/119.01)=1.4; *p*=0.23; *η* ^2^=0.055) (Figure 4). Univariate analyses however revealed a significant difference in the recovery delta between groups (*F*(2/48)=4.13; *p*=0.022; *η* ^2^=0.147). Single group comparisons revealed a significant difference between RM and R (*F*(1/31)=0.547; *p*=0.026; *η* ^2^=0.15) and between RM and SW (*F*(1/29)=4.7; *p*=0.039; *η* ^2^=0.139) in the recovery delta; sAA activity in the RM condition is back at baseline at T4 (+ 25 min), compared to R or SW at T5 (+ 40 min).

### Cardiac measures

**Figure 5:**
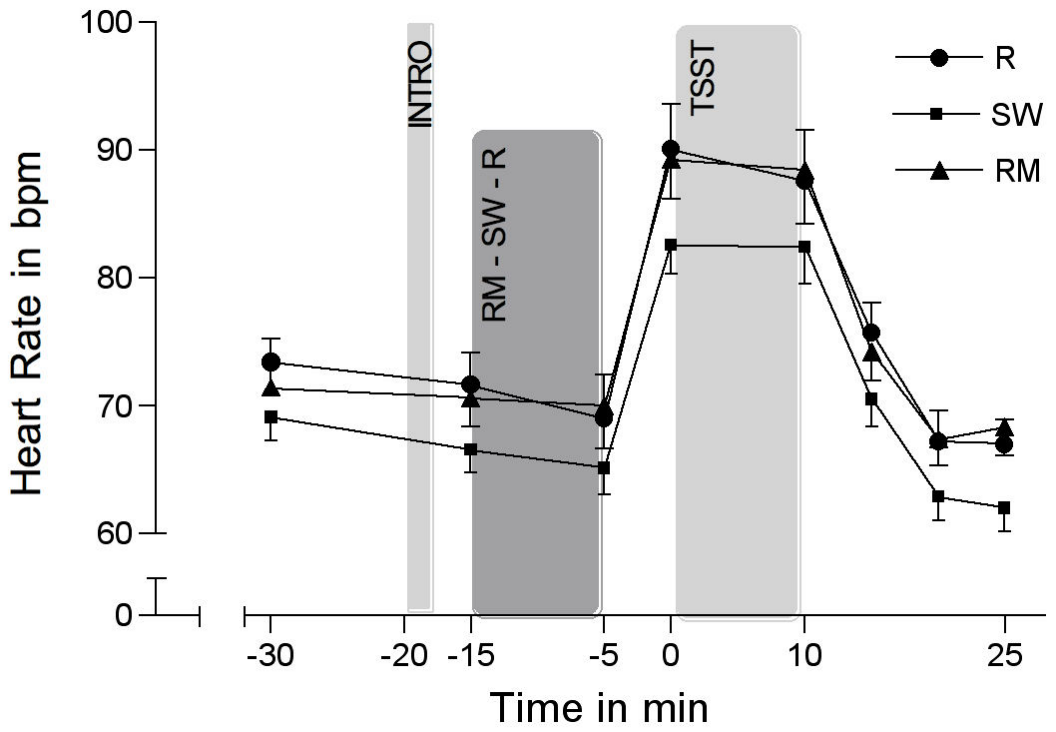
Heart rate in response to the TSST. Heart rate in response to the TSST (means ± SEM) in the experimental group listening to relaxing music (RM), the control group listening to sound of rippling water (SW), and the control group resting without acoustic stimulation (R).

**Figure 6:**
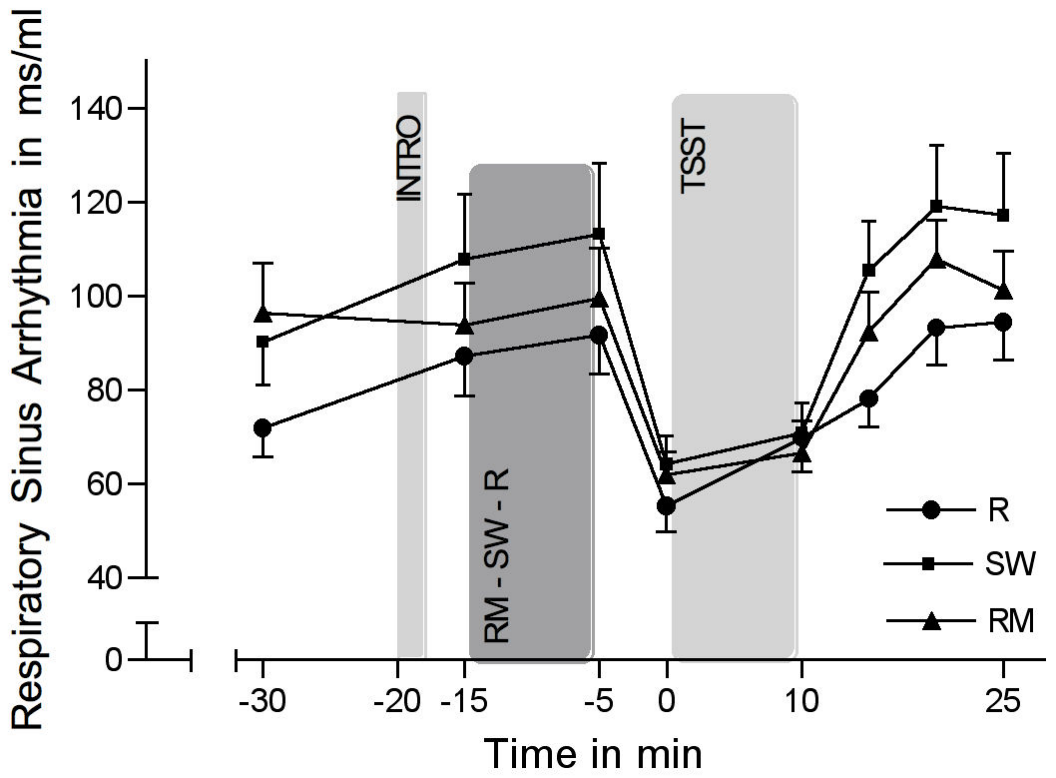
Respiratory sinus arrhythmia in response to the TSST. Respiratory sinus arrhythmia in response to the TSST (means ± SEM) in the experimental group listening to relaxing music (RM), the control group listening to sound of rippling water (SW), and the control group resting without acoustic stimulation (R).

Cardiac measures changed significantly over the course of the experiment over time (HR:F(3.16/151.77) = 122.05; *p*<0.001; *η* ^2^=0.027; RSA: *F*(3.3/158.49)=20.41; *p*<0.001; *η* ^2^=0.298). HR and RSA showed mirrored stress responses (FIGURES 5 and 6). Without the inclusion of the control variables (i.e. BDI, ERQ, STAI-trait, TICS), repeated-measures ANOVA revealed no significant group differences concerning HR (group-by-time interaction: *F*(6.32/151.77)=0.66; *p*=0.692; *η* ^2^=0.027) or RSA (group-by-time interaction: *F*(6.6/158.49)=0.86; *p*=0.533; *η* ^2^=0.035). Also with the inclusion of the control variables, groups did not significantly differ over the course of the experiment concerning HR (group-by-time interaction: *F*(5.73/103.2)=0.6; *p*=0.72; *η* ^2^=0.032) or RSA (group-by-time interaction: *F*(5.76/103.7)=0.96; *p*=0.456; *η* ^2^=0.05). However, groups significantly differed in the recovery delta of RSA (5 min after cessation of TSST) (*F*(2/40)=4.06; *p*=0.025; *η* ^2^=0.169): Single group comparisons revealed a significant difference between SW and R (*F*(1/27)=6.70; *p*=0.015; *η* ^2^=0.199), suggesting a faster recovery of SW after the TSST in RSA when compared to R.

### Psychological stress measures

The subjective perception of stress during the experiment (VAS), as well as the anxiety (STAI-state) significantly changed over time: VAS and STAI-state increased from baseline to after the Intro of the TSST, deceased in response to the experimental and control conditions (T2, -5 min), increased in response to the TSST (T3, + 10 min) and decreased again thereafter (T4, + 25 min) (VAS: *F*(2.57/141.13)=18.79; *p*<0.001; STAI-state: *F*(2.9/50)=22.55; *p*<0.001). For all further analyses, we controlled for the influence of chronic stress (TICS) when analyzing the VAS scale of the perception of stress, and we controlled for the influence of trait anxiety (STAI-trait) when analyzing the STAI-state. Neither the VAS (group-by-time interaction: *F*(5.14/136.12)=0.79; *p*=0.562), nor the STAI-state (group-by-time interaction: *F*(5.79/141.87)=1.42; *p*=0.213) did significantly differ between groups.

## Discussion

The purpose of the current study was to examine the effects of listening to relaxing music prior to a laboratory stressor on endocrine, autonomic, cognitive, and emotional responses in healthy women. We hypothesized that those participants who listened to relaxing music prior to a stress task would show different stress responses in comparison to non-music control groups, i.e. an acoustic control condition (sound of rippling water) and a control condition, resting without acoustic stimulation. With regard to endocrine responses, we found significant differences between the music and the acoustic control condition in cortisol: highest cortisol concentrations were observed in individuals who were listening to music prior the stress task, lowest concentrations were found in those who were listening to the sound of rippling water. Although there was no significant effect of music regarding autonomic responses, we observed a trend towards a faster recovery in sAA activity and in RSA in the music group. As for psychological measures, we did not find significant differences between the three groups.

The lack of a decreased cortisol response in the music group corroborates findings of a previous study by Knight and Rickard [26]. To the best of our knowledge, that investigation is the only laboratory-based study in which the influence of music listening prior to a stressor was investigated. However, the explanatory power of the findings was somewhat limited as the authors did not apply a stressor that was strong enough to elicit a significant cortisol stress response and that participants were examined in small groups (6–12 participants per condition). As for the latter, recent research has suggested that music interventions might be more effective on an individual compared to the group level [54]. Nevertheless, even though we applied a significant stressor and tested our participants one-by-one, we did not find an attenuation of stress-induced cortisol levels after music listening.

We did not expect the observed relative increase of cortisol concentrations in the music group in comparison to the control groups. Our findings seem to be the opposite from what the majority of previous studies have reported when investigating the effect of music on *baseline* HPA axis functioning, i.e. a significant decrease in cortisol concentrations [67–77]. Our findings also seem to differ from the effects of music, when music is presented *during* (suppressed cortisol response) [73] or *after* a stressor (decreased cortisol response) [5]. Interpreting our findings in the light of these studies, we may deduce that the beneficial effect of music on HPA axis functioning depends on situational context (rest vs. stress) and chronology of events (prior vs. during vs. after stress). A possible explanation for the context-dependent influence may be the involvement of certain brain areas and their subdivisions, such as for instance the hypothalamus, the amygdala, the hippocampus and the prefrontal cortex, in both listening to music and stress processing [8,34,36,78–80]. It appears that music listening prior to the experience of stress may add to, facilitate, or increase subsequent HPA axis activation by a staggered activation of a partly shared neuronal network. Brain-imaging studies are needed to investigate whether there is indeed a specific ‘combinational’ effect of music and stress.

It is of great interest that the lowest concentrations of cortisol were observed in the acoustic control condition (i.e. listening to the sound of rippling water). Given that, to the best of our knowledge, no study so far has investigated the effects of natural sounds on stress-related HPA axis responses, we did not anticipate this outcome. The sound of rippling water was equally preferred and perceived as relaxing as music by our participants. This may be a hint to differential effects on the HPA axis of music and non-music acoustic stimulation. Due to the lack of studies on neuronal activation patterns of natural sounds it is difficult to determine the exact mechanisms for this effect, though. Although unexpected, the finding of decreased cortisol concentrations in the acoustic control condition is of potentially great relevance and may increase our understanding of mechanisms of beneficial interventions in natural environments, based on the concept of biophilia for instance [81,82]. According to this view, humans, who have lived in natural environments throughout evolution, are equipped with brains / mental functioning that “…subconsciously seek connection with all that is alive and vital” (p. 4660) [81]. As a consequence, humans indeed feel more comforted in natural than in urban environments. Lingering in natural environments, such as for instance in a forest, has been found to have significant beneficial physiological effects [83]. Accordingly, a detachment from natural environments might lead to decreased physical and psychological well-being [84]. It might therefore be reasoned that the sound of rippling water in our study had a relaxation effect stronger than that of music, due to its inherent characteristics as a sound of nature. Of course, this notion is highly speculative. More studies are needed to examine the differential endocrine effects of natural and non-natural acoustic sound stimulation.

We observed a differential influence of music listening on autonomic activity: music resulted in a faster autonomic recovery after stress compared to the control groups. This partly corresponds with findings from an investigation by Arai et al. [85] who found significantly decreased sAA levels at wound closure in patients who listened to intra-operative music when compared to a non-music control condition. Music might thus facilitate autonomic recovery from a stressor in comparison to listening to non-musical sounds or no acoustic stimulation. The fact that our finding only showed a statistical trend narrows its relevance, however. Other investigations assessing the effects of music on the ANS (e.g. via epinephrine and norepinephrine) have found no beneficial effects [37,86]. As for cardiac measures, we found a decrease in HR and an increase in RSA in response to RM, SW, and R. After stress exposure, we found an increase in HR and a decrease in RSA. On the one hand, these findings correspond to investigations that found an increase of parasympathetic activity in response to sedative music listening [87–89]. On the other hand, our results corroborate findings from studies reporting decreased parasympathetic activity in response to stress [90,91]. As with sAA, we found a trend for a faster recovery of the RSA in the music group when compared to the resting control group. It appears that music listening might be effective in accelerating the recovery process of the parasympathetic branch of the ANS. It is interesting, however, that the sound of rippling water was even more effective than music in returning RSA levels back to baseline. Clearly, further studies are warranted for further eliciting the differential physiological effects of music and non-music acoustic stimulation.

Music listening had no differential effect on psychological measures (stress perception or anxiety) in comparison to the two control conditions. This is not in line with investigations that report listening to music to be effective in reducing psychological stress [33] or anxiety [26,37–39]. One explanation might be that music listening may only reduce psychological stress / anxiety in the presence of a relatively mild stressor. It might be the case that the stressor in our study (i.e. the TSST) was too strong. Knight and Rickard [26], who were using a (mild) cognitive stressor in the laboratory, found anxiety-reducing effects of music listening prior to stress. MacDonald and colleagues found similar effects only in those patients who had a minor surgery (mild stressor) and not in those who had a major surgery [92]. Evans [40], finally, systematically reviewed studies of the effectiveness of music interventions for hospital patients. He found that music listening was effective for the reduction of anxiety during normal care delivery (which may be considered as mild stressors), but not for patients undergoing invasive or unpleasant procedures (strong stressors). In contrast to those findings, however, patients in the study by Allen et al. were experiencing “…a high level of stress and anxiety...” [33] related to surgery, so that one may assume that this was a strong stressor. Still, music was effective in decreasing perceived stress levels in that study. However, patients were allowed to listen to their own choice of music. It might be argued that not the music itself, but the positive memories associated with it caused this effect. What is more, control patients did not wear headphones and were therefore exposed to the sounds of surgery, thus further inducing stress in the control group. Future studies are needed to test for the assumption that music listening might only reduce stress related psychological processes and anxiety in the context of mild stressors.

Taken together, our results seem to indicate that pre-stress music listening might not be effective in reducing the biopsychological stress response, but might, in contrast, add to or facilitate a stress response. However, our results may also be interpreted in the light of another explanation: it may be that the participants in the music group were actually so relaxed that the subsequent stress induction was incompatible with this state of relaxation, and that they produced an increased stress response as a consequence. We might have therefore measured the effect of the contrast between a relaxing and a stressful state rather than the preparatory effects of relaxing music on the subsequent stress response. This notion is supported by the greatest increase in stress perception in the relaxing music group. Future studies should follow-up on this explanation and further dissect the effects of preparatory music listening on stress responses.

Although this is the first study in which the effect of pre-stress music listening on a multitude of stress response domains was examined in the context of a rigorously controlled laboratory setting, our findings need to be considered in the light of the following limitations.

### Selection of music stimulus

Standardized music stimuli, selected by the researchers, might have different effects than those chosen by the participants themselves. In our study, however, we used a music stimulus which had already been evaluated as relaxing in previous research [6], so we were confident that this stimulus had stress-attenuating capacity independent of individual preferences. Also, using researcher-selected music stimuli has been shown to have greater effects on stress reduction than music stimuli selected by the subjects themselves [54].

### Sample

The focus on healthy young female participants, which were non-smoking, not taking any oral contraceptives, and being in the follicular phase of the menstrual cycle, restricts the generalization of the results beyond this particular sample. While the exclusion of potential confounding variables certainly improves internal validity, further research should investigate men also, as well as a mixed sample of men and women with more liberal inclusion criteria in order to reach more general conclusions.

In summary, the findings of the present study demonstrate that listening to relaxing music prior to a stress task differentially affects biological stress response domains. Listening to relaxing music prior to a stressor did not decrease the endocrine stress response, but tended to increase it. Moreover, music listening helped the ANS to recover from a stressor more efficiently. Cognitive and emotional processes did not seem to be differently influenced by listening to relaxing music compared to listening to the sound of rippling water or resting with no acoustic stimulation prior to a stressor. As a consequence, our findings do not fully support the notion of using music listening as a successful stress management tool, at least not in the context of anticipating an upcoming stressor. Certainly, the potential health implications of the observed increase in HPA axis activation and the faster recovery of the ANS through listening to relaxing music are worth mentioning and should be further studied in order to better understand the potentially positive effects of music on the human body.
